# Prevalence of work-related musculoskeletal disorder and ergonomic risk practice among medical laboratory professionals at health facilities of eastern Ethiopia

**DOI:** 10.3389/fpubh.2024.1443217

**Published:** 2024-12-19

**Authors:** Dagim Fikre, Behailu Hawulte Ayele, Akewok Sime, Fikru Tebeje, Fitsum Weldegebreal

**Affiliations:** ^1^Bisidimo General Hospital Health Science College, Bisidimo, Ethiopia; ^2^School of Public Health, College of Health and Medical Sciences, Haramaya University, Harar, Ethiopia; ^3^School of Medical Laboratory Sciences, College of Health and Medical Sciences, Haramaya University, Harar, Ethiopia; ^4^Laboratory Bacteriology Research, Department of Diagnostic Sciences, Faculty of Medicine and Health Sciences, Ghent University, Ghent, Belgium

**Keywords:** ergonomics, musculoskeletal disorder, medical laboratory professionals, Ethiopia, prevalence

## Abstract

**Background:**

Musculoskeletal disorders represent a significant occupational problem due to poor ergonomic workstations among medical laboratory professionals; however, there is limited information regarding ergonomic-related musculoskeletal disorders among laboratory personnel in Ethiopia, particularly in eastern Ethiopia.

**Methods:**

An institutional-based cross-sectional study design was implemented among 241 Medical Laboratory Professionals (MLPs) from December 20, 2023, to January 20, 2024. A standardized questionnaire adapted from the Nordic musculoskeletal questionnaire and a combination of self-administered surveys and direct observational techniques was used for data collection. Bivariate and multivariable logistic regression analyses were used to determine factors associated with musculoskeletal disorders with findings presented through Odds ratios and a 95% Confidence Interval (CI), and statistical significance was declared at *p*-value <0.05.

**Result:**

This study showed that over the past 12 months, 142 (58.9%; 95% CI: 52.0, 65.0) Medical Laboratory professionals reported work-related musculoskeletal disorders at least in one of the nine body parts. Age 36 and above years (AOR = 1.51; 95% CI: 1.02, 6.01), being female (AOR = 1.89; 95% CI: 1.09, 5.04), work experience 10–15 years (AOR = 3.99; 95% CI: 1.6, 9.4), work experience >15 years (AOR = 4.13; 95% CI: 1.52, 10.81), sitting time 4 and above hours (AOR = 2.25; 95% CI: 1.10, 4.63), patient load >300 (AOR = 2.67; 95% CI: 1.12, 7.46), and being overweight (AOR = 1.67; 95% CI: 1.04, 6.03) were factors significantly associated.

**Conclusion:**

The prevalence of work-related musculoskeletal disorders was found to be higher among Medical Laboratory Professionals as compared to previous research conducted in Ethiopia. It is recommended to regularly evaluate workstations to reduce strain through adjustments in the chair and bench heights and implement task rotation to reduce repetitive movements and distribute physical demands among staff, in addition to that, adjust work schedules to include intermittent breaks. Tailored ergonomic solutions and flexible arrangements should be provided for female professionals who are at a higher risk for work-related musculoskeletal disorders. Furthermore, conducting routine health screenings to detect early signs of musculoskeletal disorders for timely intervention, as well as enhancing awareness among Medical Laboratory Professionals, are essential measures to mitigate ergonomic risk practice.

## Introduction

Musculoskeletal disorders (MSD) are pathological conditions impacting muscles, nerves, tendons, joints, cartilage, and spinal discs, frequently associated with occupational risk factors (1). Among the leading causes of long-term disability and illness are musculoskeletal disorders (MSDs), which are closely related to functional disability and, consequently, to high expenditure on health and social resources (2). Musculoskeletal Disorders are typically characterized by pain and temporary or lifelong limitations in mobility and dexterity, which reduce people’s ability to work and participate in social life (3). To date, work-related MSDs are among the major risk factors practices for the occurrence of certain diseases, such as osteoarthritis, osteoporosis, and sarcopenia, and conditions that also affect multiple body areas or systems, such as a regional pain state and inflammatory diseases (2). These pathological conditions are considered major diseases affecting millions of workers, resulting in a cost of billions to companies and public health systems (4).

Globally, approximately 1.71 billion individuals, including laboratory personnel, are affected by musculoskeletal disorders (MSDs), leading to enduring pain, diminished productivity, and escalated healthcare expenditures (5) and the Global Burden of Disease (GBD) study has identified MSDs as the second most prevalent cause of disability worldwide (6). In accordance with the systematic review and meta-analysis conducted by Reza Tavakkol et al. regarding the prevalence of musculoskeletal disorders (MSDs) among operating room staff, it was determined that the incidence of such disorders in the neck, shoulders, elbows, wrists and hands, upper back, lower back, hips, knees, and ankles and feet was measured at 53.66% (7).

Healthcare professionals are recognized to face an increased susceptibility to Musculoskeletal Disorders (MSD), with a prevalence rate in Nigeria varying from 26 to 69%. This escalated risk can be attributed to ergonomic risk factors practices, resulting in a substantial prevalence of MSDs among Medical Laboratory Professionals (MLPs) (8). Laboratory workers are susceptible to MSDs because of repetitive and frequently strenuous tasks such as manipulating microscopes, pipetting, and handling hazardous chemicals (6, 9). According to Chia et al., 95% of medical technologists reported encountering WMSDs in at least one body location. There was a connection observed between diminished work capacity and the presence of WMSDs impeding daily activities (10).

A research study carried out in governmental health laboratories in Addis Ababa, Ethiopia in 2012 revealed an insufficient ergonomic layout of workstations. Moreover, the study links MSD complaints with uncomfortable desks, with Ankle/feet problems representing 21.7%, and Knees being the most prevalent (20.8%). A notable factor contributing to the increased risk of WMSD development among MLPs is the lack of compliance with ergonomic principles (11). Importantly, to attain effective and efficient results, the health and safety of medical laboratory workers need to be prioritized, as 60–70% of decisions concerning patient admission, medication prescriptions, and discharges are reliant on the collaboration of medical laboratories (12).

Several factors contribute to the onset and progression of these disorders, including predisposing genetic and environmental factors (13). Furthermore, work-related musculoskeletal disorders (WMSD) exhibited a correlation with the demographics of physical therapists, including their age, gender, area of specialization, and specific job responsibilities. Engaging in manual therapy, as well as the lifting and transferring of patients, constitutes tasks frequently linked to the onset of WMSD among physical therapists (14). The latter include workplaces and the activities that are performed in these settings, where we spend most of our daily lives. There is a notable shortage of healthcare professionals in Ethiopia, specifically in the medical laboratory sector, resulting in a potential rise in workload and subsequent emergence of Work-Related Musculoskeletal Disorders (WMSDs) (15). A research gap exists in the understanding of the extent of musculoskeletal disorders (MSDs) related to ergonomics and the factors associated with them among medical laboratory workers in Ethiopia. So, this study aims to provide a comprehensive assessment of the impact that different work-related activities have on the musculoskeletal system. It seems crucial to better frame this issue and its causes, especially in the workplace, given the seemingly continually growing trend of MSDs, to develop effective and lasting prevention strategies in countries like Ethiopia where a notable shortage of healthcare professionals with high workload presents. so, this study was specifically conducted on medical laboratory professionals, a group often neglected in ergonomic studies, contrasting with prior research that focused on healthcare workers or office settings and it provided a comprehensive assessment of ergonomic practices and its implications for local health policy and future research. Hence, the primary objective of this study was to examine the prevalence of ergonomics-related MSDs and their associated factors among medical laboratory professionals in Ethiopia.

## Methods and materials

### Study area and period

This study was conducted in the Harari region and Dire Dawa City administration health facilities from December 20, 2023, to January 20, 2024. According to the report by the Dire-Dawa Health Bureau, during the surveying time, the Dire Dawa City administration had 2 public hospitals (Dill Chora Hospital and Sabian Hospital), 15 health centers, and 35 health posts. Apart from these, there were 1 Hospital, 3 primary Hospital, 32 medium clinics, 7 primary clinics, 17 pharmacies, 53 drug stores, and 3 diagnostic laboratories possessed by private owners (16). The study included two public hospitals (Dill Chora Hospital and Sabian Hospital), five private hospitals, and 18 owned private medium and specialty clinics. The Harar region has two public hospitals (Hiwot Fana Comprehensive Specialized University and Jugol General Hospital), one Federal Police Hospital, one private General hospital, eight government health centers, 34 health posts, and one Family Guidance Association (17). In the study, the two public hospitals, Harar General Hospital, and Federal Police Hospital, in addition, to ten government-owned health centers participated.

### Study design and population

An institution-based cross-sectional study design was employed. During the study period, 241 Laboratory professionals who had fixed working hours and engaged in diagnosing health facility laboratories in the private and government health facilities of the Harari region and Dire Dawa city administration were included in this study.

#### Inclusion criteria

Medical Laboratory professionals, who were engaged in the diagnosis of health facility laboratories who have fixed working hours.

#### Exclusion criteria

Medical Laboratory professionals, who worked less than 1 year of work experience, who were on annual leave and maternal leave, and an individual who had a history of accidents outside of their workplace affecting the musculoskeletal system.

### Sample size determination and sampling technique

The sample size of the study was determined utilizing a single population proportion formula, considering the previous prevalence of WRMD in Addis Ababa reported as 66.6% (18). This computation was conducted with a 95% confidence interval (CI), a 5% margin of error, and a 10% non-response rate. As a result, the determined sample size was 213, closely resembling the total number of MLPs working in governmental and private health institutions in the Harari region and Dire Dawa city, which was 245. The discrepancy between the specified sample size and the actual number of MLPs was minimal. Therefore, the study included all laboratory personnel who met the defined criteria from health facilities in the Harari region and Dire Dawa city administration, selected through a convenient sampling method.

### Data collection methods and procedures

Data was collected by a self-administered standardized Nordic questionnaire and facility observation checklists by four MLPs and supervised by two senior MLPs. A modified Nordic questionnaire was used to measure the symptoms of the musculoskeletal disorder (19) and this questionnaire includes socio-demographic characteristics, individual factors such as physical exercise, nutritional status, awareness about MSDs, and behavioral factors such as cigarette smoking, shisha smoking, alcohol drinking, chat chewing, and history with systematic illness and ergonomic risk practice factors such as a workstation, awkward position, repetitiveness, working hour, break between work, the average number of hours, standing per day, the average number of hours sitting per day, overtime work, housework and Nordic Musculoskeletal Questionnaire, which has a Nordic measurement that divides the human body into nine body areas (20). Facility-based direct observation checklists were used to assess perceived safety, adequacy, and appropriateness of workplace medical laboratory workstations such as laboratory bench, computer workstations, pipetting, microscope, chair, and workstation conditions. For the laboratory workstation such as safety and sufficiency of medical laboratory workstations, which include laboratory bench, workstation, pipetting, micromanipulation, and computer station were assessed and recorded as strongly disagree (1-point), disagree (2-points), neutral (3-points), agree (4-points) and strongly agree (5 points) based on their perception level. Surveyed data was gathered at the chosen workstation while employees of the Medical Laboratory Professional were at their jobs. When a worker experiences pain in at least one of nine body parts, it is referred to as a musculoskeletal disorder (21, 22). By integrating these elements, the study aims to provide a comprehensive understanding of how various factors contribute to the prevalence and impact of pain associated with WMSDs among medical laboratory professionals. This holistic approach helps identify specific areas for intervention and improvement in workplace ergonomics and health practices.

### Methods of data analysis

The collected data was checked, edited, coded, and entered into EPI-Data version 3.1 and exported to Statistical Package for Social Science version 26 for additional analysis. WMSD data was obtained for the preceding 12 months, 1 month, and 7 days. Annual prevalence was chosen in this study because it was a suitable time scale similarly practiced in previous works.

The application of descriptive statistical analysis was employed to describe the socio-demographic and clinical attributes of the study participants, as well as the prevalence of Work-Related Musculoskeletal Disorders (WMSDs). To assess the perceived safety, adequacy, and appropriateness of medical laboratory workstations, including laboratory benches, workstations, pipetting, micromanipulation, and computer stations, a 5-point Likert scale was employed, ranging from strongly disagree (1-point) to strongly agree (5-point).

The mean score for safety and adequacy of medical laboratory workstations was ascertained by computing the average based on the perceived levels of safety and sufficiency. The computation of overall satisfaction utilizing the Likert scale entailed multiplying the count of strongly agree ratings by 5, agree ratings by 4, neutral ratings by 3, disagree ratings by 2, and strongly disagree ratings by 1, then dividing the total by the number of ratings (1–5) for ergonomic risk practices. A mean score exceeding 4 signified an optimal perception of the safety and adequacy of workstations utilized for tasks within the medical laboratory environment. The relationship between different factors and Work-Related Musculoskeletal Disorders (WMSDs) was explored through bivariable and multivariable logistic regression analyses. All explanatory variables showing significance at a level of *p* ≤ 0.25 in the bivariable analysis were integrated into the multivariable logistic regression model. Variables indicating *p* < 0.05 in the multivariable analysis were considered statistically significant.

### Data quality assurance

Data quality was ensured in different ways first, questioners were translated into the local language (Amharic and Afaan Oromo), and check for health professionals familiar with the medical terminologies made the forward translation of the Extended Nordic musculoskeletal questionnaire from English to the local language (Amharic and Afaan Oromo). Instead of literal equivalence of the terminologies, the approach in the translations emphasized cross-cultural translations; subsequently, reverse translation into English was done by an expert in the English language. First, 2 days of training on data collection instruments and data collection techniques were given to the supervisor and data collectors. 5% of the sample was been pre-tested at Bisidimo General Hospital which was outside the study site before actual data collection to ensure its consistency and evaluate their clearness and applicability according to the objective of the study. The Nordic musculoskeletal questionnaire had an anatomical diagram of nine body regions like neck, shoulder, upper and lower back, hands/wrists, arms, knee, thighs, and feet to make it easy for study participants to correctly identify the presence of musculoskeletal symptoms. The tool was finalized after the pre-test and necessary modifications. In the final analysis, data from the pre-test was not included. The supervisor oversaw the data collectors daily, and the researchers checked all filled-out formats for completeness during data collection. Finally, the supervisor and investigator ensured that all acquired data were complete and consistent during data management, storage, and analysis.

### Ethical considerations

Ethical clearance was obtained from Haramaya University’s College of Health and Medical Science Ethical Review Committee (Ref. No. IHRERC/244/2023). Letters of cooperation were then written to each health facility. The purpose of the study was explained to the clinic and hospital head and each study participant, including the objective procedures, potential risks, and benefits of the study. The study participants were informed of their ability to decline or withdraw from the study at any time, and refusing to participate did not affect any of them. Participants’ confidentiality was secured by excluding names and identifiers from the questionnaires. Throughout the study, the clinic and hospital’s head and respondents provided informed, voluntary, written, and signed consent.

## Results

### Socio-demographic characteristics

Of the total 245 medical laboratory professionals, 241 were included in this study with a response rate of 98.3%. Males made up 108 (37.3%) of the total. The age range of the study participants was 22 to 56 years, with a mean (standard deviation (SD)) of 32.2 (±6.7) years. The majority of participants, or 72.6%, were between the ages of 20 and 35. The majority of respondents—133, or 55.2%—were married, and 89, or 36.9%—worked in government-owned healthcare facilities. The majority of participants—192, or 79.7%—had a first degree, while 81, or 33.6%, had less than 5 years of professional experience. Regarding the salary distribution, 163 respondents (67.6%) made more than 7,501 Ethiopian Birrs per month; however, after accounting for additional income and duty over 3 months, 106 respondents (43.6%) made less than 10,000 Ethiopian Birrs (Table 1).

**Table 1:** Socio-demographics characteristics of study participants in Dire Dawa city and Harar, eastern Ethiopia, 2024 (*n* = 241).

Variables	Categories	Frequency	%
Gender	Male	138	57.3
	Female	103	42.7
Age	22–32	138	57.3
	33–43	85	35.3
	>43	18	7.5
Marital status	Single	97	40.2
	Married	133	55.2
	Widowed	8	3.3
	Divorced	3	1.2
Educational level	Diploma	47	19.5
	Degree	192	79.7
	Master	2	0.8
Experience	1–5	81	33.6
	5–10	70	29
	10–15	53	21.8
	>15	37	15.3
Monthly salary	<4,501	5	2.1
	4,501–7,501	73	30.3
	>7,501	163	67.6
Institution	Governmental hospital	89	36.9
	Health center	70	29.0
	Private hospital	36	14.9
	Private clinic	32	13.3
	Others^a^	14	5.8
Other income^b^	<10,000	105	43.6
	10,000–20,000	69	28.6
	>20,000	67	27.8
Employment status	Permanent	221	91.7
	Contract	20	8.3

a Blood bank, Child Health and Mortality Surveillance (CHAMPS), Atlas laboratories, International clinical laboratories.

b Average duty and other income for 3 months.

### Organizational and behavioral-related factors

One hundred sixty-nine (70.1%) of participants worked more than 40 h per week and 112 (46.45%) dealt with 301–1,000 patients per week. About 111 (46.1%) MLPs were assigned to miscellaneous laboratory service sections and 161 (66.8%) of MLPs did not know about ergonomics. Around 50 (20.7%) of medical laboratory professionals had second jobs and 172 (71.3%) worked overtime. Around 163 (67.6%) of the participants had housework. One hundred ninety-eight (82.2%) of medical laboratory professionals were sitting in the workplace for 1 to 3 h daily and 127 (52.7%) average standing time was 1 to 3 h. About 81 (33.6%) MLPs were current alcohol users and 89 (36.9%) were current khat chewers. A small number (4.1%) were current shisha consumers. Close to three-fourth, 73.9% of the participants had normal BMI (18.5–24.9 kg/m^2^) (Tables 2, 3).

**Table 2:** Organizational factors associated with WMSDs of MLPs working in Dire Dawa city and Harar, Ethiopia, 2024 (*n* = 241).

Variables	Categories	Frequency	Percentage
Working hours per day	= < 40	169	70.1
	>40	72	29.9
Second job	Yes	50	20.7
	No	191	79.3
Overtime	Yes	172	71.3
	No	69	28.7
Housework	Yes	163	67.6
	No	78	32.4
Average standing time per day	1–3 h	127	52.7
	4–6 h	114	47.3
Sitting time per day	1–3 h	198	82.2
	4–6 h	43	17.8
Number of patients served per week	<150	18	7.5
	151–300	111	46.05
	301–1,000	112	46.45
Laboratory section	Clinical chemistry	25	10.4
	Hematology	22	9
	Bacteriology	26	10.8
	Parasitology and urinalysis	32	13
	Sample collection room	25	10.4
	Miscellaneous	111	46.05

**Table 3:** Behavioral factors associated with WMSDs of MLPs working in Dire Dawa city and Harar, Ethiopia, 2024 (*n* = 241).

Variables	Categories	Frequency	Percentage
Ever alcohol	Yes	81	33.6
	No	160	66.4
Current alcohol use	Yes	57	70.3
	No	24	29.7
Ever Khat use	Yes	89	36.9
	No	152	63.1
Current khat chewer	Yes	80	89.8
	No	9	
Ever Cigarette smoker	Yes	19	7.9
	No	222	92.1
Current cigarette smoking	Yes	19	100
	No		
Ever Shisha use	Yes	10	4.1
	No	231	95.9
Current shisha use	Yes	3	30
	No	7	70
Nutritional status	Underweight (<18.5)	16	6.6
	Normal (18.5–24.9)	178	73.9
	Overweight (>24.9)	47	19.5

### Previous medical history

About 142 (58.9%) medical laboratory professionals experienced WMSD, and 90 (44.6%) of participants reported that the cause of the disorder was uncomfortable body positions (awkward work environment). Only 50 (35.2%) visited the doctor for WRMSDs and 40 (80%) took medication. Twenty-three (46%) took sick leave due to WRMSDs for 3 to 15 days, with an average of 7.52 days. Additionally, half of them were currently receiving medication for their condition (52%). The majority (96.7%) reported experiencing symptoms related to WMSDs after starting their current job (Table 4).

**Table 4:** Past medical history of MLPs working in Dire Dawa city and Harar, Ethiopia, 2024 (*n* = 241).

Variables	Categories	Frequency	Percentage
Have you ever experienced WMSD	Yes	142	58.9%
	No	99	41%
What was likely cause of pain	Repetition	60	29.7
	Awkward	90	44.6
	Vibration	10	4.9
	Static loading	40	19.8
	Forceful exertion	2	1
Visit a doctor for WRMSD diagnosis	Yes	50	35.2
	No	92	64.8
Types of diagnosis	X-Ray	28	56
	MRI	3	6
	CT Scan	1	2
	Clinical diagnosis	18	36
Types of medication	Medicine	40	80
	Advise	3	6
	Physio therapy	7	14
Sick leave	Yes	23	46
	No	27	54
Currently taking medication for WRMSD	Yes	26	52
	No	24	48
Types of medication	Medicine	23	88.5
	Physiotherapy	3	11.5
Symptom related to WMSDs before engaging in the current work	Yes	8	3.3
	No	233	96.7
Medical history of systematic illness	Yes	34	14.1
	No	207	85.9
Chronic medical problem	Yes	17	7.1
	No	224	92.9

### Prevalence of work-related musculoskeletal disorder

**Figure 1:**
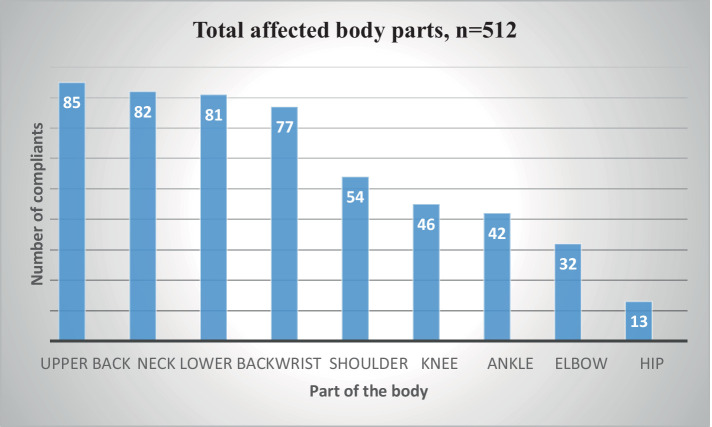
Prevalence of WRMSD in nine body parts among medical laboratory professionals working in Dire Dawa city administration and Harari regional state health facilities, 2024 (*n*=241).

After computing WRMSD at nine body parts, overall, 142 (58.9%; 95% CI; 52, 65%) of the respondents had WRMSD and they reported 512 affected body parts. The most affected body parts were the upper back (85 (35.3%)), followed by the neck (82 (34%)) and lower back (81 (33.6%)) (Figure 1).

### Prevalence of WMSDs in private vs. governmental health facilities

MLPs who were working in governmental health facilities reported a higher magnitude of WMSDs (359 (71.4%)) when compared to private facilities (90 (17.8%)) based on affected body parts. MLPs who were working in the health center showed a relatively higher magnitude of WMSDs (198 (38.4%)) compared to the government-owned hospitals (166 (33%)). MLPs who were working in the medium and specialty clinic (54 (10.7%)) reported a slightly higher magnitude of WMSDs when compared to private hospitals (36 (7.1%)) based on affected body parts.

MLPs who were working in private health facilities reported a higher magnitude of WMSDs (38 (55%)) when compared to governmental health facilities (90 (37%)) and governmental hospitals 42 (47%) were less as compared to Health Centers 48 (68%). MLPs who were working in private clinics reported a higher magnitude of WMSDs (25 (78%)) when compared to private hospitals (13 (36%)). There was no statistically significant difference between the MLPs who worked in government facilities and private health facilities with a magnitude of WMSD (χ2 = 27.93, *p* = 0.55) (Table 5).

**Table 5:** Work-related musculoskeletal disorder between private and governmental health facilities among MLPS, Dire Dawa city administration, and Harari regional state private and governmental health facilities, 2024 (*n* = 241).

WMSD	Types of health facility				
	Governmental		Private		Others
	Hospitals	Health centers	Hospitals	Medium and specialty clinics	
	*N* (%)	*N* (%)	*N* (%)	*N* (%)	*N* (%)
Neck	20 (24.3)	29 (35.3)	8 (9.8)	13 (22)	12 (14.6)
Upper back	32 (37.6)	32 (37.6)	9 (10.6)	2 (2.4)	10 (1.2)
Lower back	28 (34.6)	30 (37)	3 (3.7)	7 (8.6)	13 (16)
Shoulder	17 (31.5)	27 (50)	3 (5.5)	2 (3.7)	5 (9.2)
Ankle	17 (40.5)	14 (33.3)	–	3 (7.1)	8 (19)
Wrist	18 (23.4)	20 (26)	8 (10.4)	17 (22)	14 (18.2)
Knee	17 (37.7)	20 (44.4)	2 (4.4)	6 (13.3)	1 (2.2)
Hip	6 (46.1)	6 (46.1)	–	1 (7.7)	–
Elbow	11 (34.3)	15 (48.8)	3 (9.3)	3 (9.3)	–
Total	**166 (33)**	**193 (38.4)**	**36 (7.1)**	**54 (10.7)**	**63 (10.5)**

### Factors associated with work-related musculoskeletal disorder

The multivariable logistic regression analysis revealed that age, gender, work experience, average daily sitting time, number of patients served per day, and body mass index (BMI) were all statistically significant factors associated with Work-Related Musculoskeletal Disorders (WRMSDs), with a *p*-value of less than 0.05. Female medical laboratory professionals (MLPs) were found to be 1.89 times more likely to acquire WRMSDs compared to their male counterparts (AOR = 1.89; 95% CI: 1.61–8.04) and MLPs aged 36–60 exhibited a 1.51 times higher likelihood of developing WRMSDs than those aged 23–35 (AOR = 1.51; 95% CI: 1.02–6.01). Laboratory personnel with more than 15 years of experience had a 4.1 times higher risk of developing WRMSDs (AOR = 4.1; 95% CI: 1.5–10.8). Average sitting time per day in the workplace was also significantly associated with WRMSDs. MLPs who sat for 4 or more hours daily had an AOR of 2.25 (95% CI: 1.10, 4.63), highlighting the impact of prolonged sitting.

MLPs who served over 300 patients per week 2.67 times (AOR = 2.67; 95% CI: 1.12–7.46) were more likely affected by WRMSDs compared to those who handled a few patients. Being overweight had an AOR of 1.67 (95% CI: 1.04, 6.03), suggesting a link between BMI and WRMSDs (Table 6).

**Table 6:** Bivariate and multivariable analysis to assess the factors associated with WRMSD in Dire Dawa city administration and Harari regional state health facilities, eastern Ethiopia, 2024.

Variable	Category	WRMSD		COR (95%CI)	AOR (95%CI)	*p*-value
		Yes	No			
		No (%)	No (%)			
Gender	Male	67 (48.6)	71 (51.4)	1	1	
	Female	75 (72.8)	28 (27.2)	2.84 (1.64, 4.91)	1.89 (1.09, 5.04)	**0.000**
Age	20–35 Yrs.	91 (52)	84 (48)	1	1	
	36–60 Yrs.	51 (77.2)	15 (22.7)	3.14 (1.64, 6.00)	1.51 (1.02, 6.01)	**0.019**
Work experience	1–5 Yrs.	31 (38.2)	50 (61.7)	1	1	
	5–10 Yrs.	47 (55.2)	23 (44.7)	3.2 (1.68, 6.4)	2.89 (1.32, 6.3)	0.007
	10–15 Yrs.	37 (66)	16 (34)	3.70 (1.7, 7.8)	3.99 (1.6, 9.4)	**0.002**
	>15 Yrs.	27 (72.9)	10 (27.1)	4.35 (1.8, 10.2)	4.1 (1.5, 10.8)	**0.004**
Do you have any housework	No	40 (51.3)	38 (48.7)	1	1	
	Yes	102 (62.6)	61 (37.4)	1.59 (0.92, 2.74)	1.06 (0.51, 2.19)	0.875
Average standing/ day	<4 Hours	32 (45.5)	40 (55.5)	1	1	
	> = 4 h	110 (65)	59 (35)	2.33 (1.33, 4.09)	1.15 (0.40, 3.34)	0.797
Average sitting/day	<4 Hours	53 (45.7)	63 (54.3)	1	1	
	> = 4 h	89 (71.2)	36 (28.8)	2.94 (1.20, 4.58)	2.25 (1.10, 4.63)	**0.004**
Number of patients served/week	0–200	23 (52.3)	21 (47.7)	1	1	0.005
	201–300	44 (51.8)	41 (48.2)	0.98 (0.47, 2.03)	1.27 (0.48, 3.33)	0.628
	>300	75 (67)	37 (33)	1.85 (0.91, 3.77)	2.67 (1.12, 7.46)	**0.007**
Nutritional status	Normal	71 (50.4)	70 (49.6)	1	1	0.000
	Under weight	10 (50)	10 (50)	0.99 (0.39, 2.52)	0.91 (0.29, 2.80)	0.866
	Overweight	61 (76.2)	19 (23.8)	3.17 (1.72, 5.84)	1.67 (1.04, 6.03)	**0.000**
Physical exercise	HEPA	48 (63.2)	28 (36.8)	1	1	
	IPA	94 (57)	71 (43)	0.77 (0.44, 1.35)	0.59 (0.28, 1.26)	0.176

The bold values in table indicated that factors significantly associated with work-related musculoskeletal disorder.

### Ergonomics risk practices

About 73% of the participants indicated that their computer station was poor in the facility where they were working. One hundred ninety-six (80.1%) respondents reported that the laboratory bench station in their workplace was poor. Regarding the laboratory chair station; 144 (59%) of them reported that the laboratory station was poor. Two hundred eight (86.3%) of the respondents indicated that the station of the microscope was not good in the facility where they were working. About 88% of the respondents responded that their pipetting practice was poor according to the criteria for pipetting while 70% of the respondents also reported that their micromanipulation did not meet the criteria for cap and recap (Table 7).

**Table 7:** Evaluation of ergonomic workstation medical laboratory workstations at health facilities in Dire Dawa city administration and Harari regional state health facilities, 2024 (*n* = 241).

Work station		Frequency	Percentage	Mean score
Computer station safety and adequacy	Poor	176	73.0	2.78
	Good	65	27	
Laboratory bench station safety and adequacy	Poor	193	80.1	2.81
	Good	48	19.9	
Laboratory chair station safety and adequacy	Poor	144	59.8	3.31
	Good	97	40.2	
Microscope station safety and adequacy	Poor	208	86.3	3.06
	Good	33	11.6	
Pipetting safety and adequacy	Poor	211	87.6	2.99
	Good	30	12.4	
Micromanipulation safety and adequacy	Poor	169	70.1	2.91
	Good	72	29.9	
**Overall safety and adequacy score**				2.97

### Workstations surveyed among health facilities

Out of 44 assessed facilities, 19 (43.1%) had even arrangement of the working surface area, however, significant workspaces (16 (36.3%)) were found to be limited in size, potentially restricting movements and contributing to ergonomic challenges for the professionals. Furthermore, only 26 (59%) of the observed laboratory workspaces maintained appropriately adjusted working heights of laboratory chairs relative to the workbench. In terms of ergonomic support and comfort, the observation revealed that 17 (38.6%) professionals had the opportunity to sit and rest during their work shifts. Additionally, rotation practices, which can help reduce ergonomic strain and promote musculoskeletal health were observed only in 13 (29.6.0%) of the health facilities (Table 8).

**Table 8:** Medical laboratory workstation surveyed at health facilities in Dire Dawa city administration and Harari regional state health facilities, 2024 (*n* = 241).

		Types of facility					
		Governmental hospital	Private hospital	Governmental health center	Clinic	Others	Total %
Working surface even	Yes	4	2	7	2	4	19 (43.1%)
	No	2	4	10	8	1	25 (56.9%)
Space limited for work movement	Yes	3	5	3	3	2	16 (36.3%)
	No	3	1	14	7	3	28 (63.7%)
Working chair correctly adjusted	Yes	2	6	10	4	4	26 (59%)
	No	4	0	7	6	1	18 (41%)
Working height correctly adjusted	Yes	2	3	8	2	3	18 (41%)
	No	4	3	9	8	2	26 (59%)
Possibility to sit and rest during working	Yes	2	6	3	3	3	17 (38.6%)
	No	4	0	14	7	2	27 (61.4%)
Lighting adequate for detailed work	Yes	4	6	16	10	2	38 (86.4%)
	No	1		1		3	6 (13.6%)
Rotation system	Yes	4	4	0	2	3	13 (29.6%)
	No	2	2	17	8	2	31 (70.4%)

## Discussion

This research aimed to evaluate the extent of work-related musculoskeletal disorders and ergonomic risk practices among Medical Laboratory professionals in eastern Ethiopia. The investigation revealed that within the previous 12 months, the prevalence of WRMSDs among MLPs in the study region was 58.9% (95% CI; 52–65%). Factors such as age, gender, work experience, average daily sitting time at the workplace, number of patients attended to per day, and nutritional status were found to have statistical significance at *p* < 0.05.

The findings of this study indicate a higher prevalence of WRMSDs compared to previous studies conducted in Ethiopia, such as a study in Bahir Dar, which reported with lower prevalence (48.7%) (23), and Kampala (48.3%) (24). The differences in prevalence rates may be attributed to variations in study design, sample size, and the specific work environments of the participants. For instance, the current study may have included a more diverse range of laboratory settings, leading to higher reported rates of musculoskeletal disorders.

This finding is higher than the prevalence from a study conducted in Egypt (52.04%) (25). The discrepancy could be attributed to factors such as the limited sample size, utilization of a cross-sectional analytical design, and reliance on subjective data gathered through the standardized Nordic Questionnaire (25). Nevertheless, this finding is lower than those reported in Bangladesh, Dhaka city (99.4%) (26), India (73.3%) (43), and Nigeria (71.4%) (27). These variations may be elucidated by differences in study design, organizational work environment variations, sample size, individual pain perception, geographical variation, and cultural distinctions.

The most commonly affected areas were identified as the upper back, neck, and lower back. This highlights specific regions where laboratory professionals are particularly vulnerable to musculoskeletal disorders. The prevalence of upper back pain (35.3%) in this study is lower than the findings from a study conducted in Saudi Arabia, where laboratory workers reported a prevalence of 48.5% for upper back pain (28) and the study done by Reza Tavakkol et al. 61.48% (7). The discrepancy in findings can be attributed to the nature of laboratory work, which often involves prolonged sitting, repetitive motions, and awkward postures that strain the upper back muscles. Additionally, both studies highlight the need for ergonomic interventions to mitigate these risks. The reported prevalence of neck pain (34%) aligns with findings from a study in Iran, 33.3% among laboratory workers (29), The findings of this investigation were consistent with the research carried out by Philippe Gorce et al. involving surgeons, who exhibited a prevalence of 41% (30) as well as with the study done by Suet Yeo Soo et al. among dentists, where in the prevalence ranged from 26 to 92% (31). This consistency suggests that neck pain is a common issue across different geographical locations and laboratory settings. Factors contributing to neck pain include poor workstation ergonomics, such as the height of the workbench and the positioning of equipment, which can lead to awkward neck postures during tasks like microscopy and pipetting. The prevalence of lower back pain (33.6%) in the current study is lower than the findings from a study in Egypt, where lower back pain was reported at a prevalence of 52.1% among healthcare workers (25). The incidence of lower back complaints in both studies may be due to different ergonomic challenges, such as non-adjustable chairs, prolonged sitting, uncomfortable postures, working while physically fatigued or injured, and repetitive task performance (32) and the physical demands of laboratory tasks that require bending or lifting. Furthermore, the prevalence of lower back issues could be linked to the utilization of nonadjustable chairs with inadequate back support in their workstations (33). The findings underscore the importance of addressing lower back pain through ergonomic assessments and modifications in the workplace. The least of complaints were reported at the elbow (13.2%) and hip (5.3%). This is supported by a previous study conducted in Baher Dar with elbow (7%) and hip (7%) (23). This could be due to the nature of the work, which typically involves less physical strain on these sites. Injuries on the elbow and hip are more related to heavy lifting.

The study noted that female laboratory professionals were more likely to be affected by WRMSDs compared to their male counterparts. This observation aligns with research conducted in Kampala (24). This may be attributed to differences in muscular strength and additional family responsibilities that could exacerbate muscle fatigue. Biologically, females exhibit heightened reactivity to organizational aspects of work that, in conjunction with physical stressors, give rise to MSDs (34). Moreover, the absence of consideration for anthropometric distinctions between genders in various workstations or tools compounds this issue. Furthermore, the heightened risk of MSDs among females may be elucidated by factors such as the female reproductive system, use of oral contraceptives, pregnancy, and childbirth (35).

Within this examination, MLPs aged between 36 and 60 displayed a greater susceptibility for WRMSD compared to those aged between 20 and 35 years. This result is consistent with research from Saudi Arabia (6) and a study by Edgar R. Vieira et al. on musculoskeletal illnesses among physical therapists and their work-related conditions (14).

Advancing age correlates with an increased likelihood of experiencing a decrease in bone elasticity, thereby prompting the onset of MSD symptoms (36).

In the current study, MLPs who engaged in sitting for more than 4 hours daily exhibited a higher susceptibility to WRMSD. This outcome is supported by studies conducted in Addis Ababa (18). Prolonged periods of sitting increase ligament strains and may locally impose greater loads on muscles and tendons, consequently heightening the risk of discomfort, pain, strains, and injuries linked to postural stress disorders, joint compression, and soft-tissue (muscles, tendons, and ligaments) injuries (37).

Within this investigation, individuals classified as overweight demonstrated a heightened likelihood of developing WMSD compared to those of normal weight. This finding is supported by the systematic review on risk factors for work-related muscular disorders by Bruno R da Costa et al. (38) and studies conducted in the Netherlands (34). The rationale behind this phenomenon may be attributed to an increase in BMI stemming from increased body fat mass, which can prompt adenopathy. Adenopathy denotes a condition where adipose tissue metabolism evolves into chronic inflammation or metabolic syndrome (39).

In the current study, medical laboratory professionals attending to over 300 patient’s week were approximately three times more prone to experiencing WRMSD. This outcome aligns with a study in Nigeria (40). With increased patient attendance, MLPs endure heightened workloads that require repetitive tasks, prolonged standing, and sustained pressure on muscle tissue, predisposing them to exhaustion. The accumulation of lactic acid from repeated exertions leading to pain could also contribute to the onset of WRMSD (41).

In this study, laboratory professionals with work experience of 15 years and above were 4 times more likely affected by WRMSD when compared with those with work experience is less than 5 years. This result is supported by a study done in Saudi Arabia (25). This could be justified as the newly employed laboratory professional will be younger than those with experience greater than 15 which restrains them not to actively participating in physical activity which could increase the chance of developing WRMSD due to age disparity (42).

The detailed assessment of the safety and sufficiency of Medical Laboratory workstations, which include laboratory bench, workstation, pipetting, micromanipulation, and computer station, reveals an average score of 2.976 that showed poor ergonomic condition. It is higher than the study conducted in Bahir Dar (23) and Addis Ababa (18). This could be a lack of knowledge about ergonomics and the unavailability of ergonomic purchased products (18). Also, the deviation may be due to data collection techniques and varying levels of subjectivity. In this study, participants filled out the checklist themselves. However, in the Addis Ababa (18), and Northwest Ethiopia Alibsew studies, the checklist was completed by a data collector. Overall, the study emphasizes the urgent need for targeted strategies to address the high prevalence of WRMSDs among medical laboratory professionals and enhance their occupational environment.

### Limitation of the study

The cross-sectional study design may limit its ability to determine causal relationships between variables. Furthermore, many measurement techniques or assessment procedures, such as the ergonomic evaluation checklist, may be limited in their ability to capture the entire complexity of ergonomic risk factors in medical laboratory settings.

## Conclusion

The prevalence of work-related musculoskeletal disorders (WMSDs) among medical laboratory professionals in eastern Ethiopia is notably high, with 58.9% of participants reporting symptoms in at least one anatomical area over the past year.

The prevalence of work-related musculoskeletal disorders (WRMSDs) among medical laboratory professionals is notably high at 58.9%, with the upper back, neck, and lower back being the most affected areas. Factors such as age, gender, work experience, and daily sitting time significantly contribute to the occurrence of these disorders. The findings underscore the urgent need for ergonomic interventions and improvements in workplace design to enhance the health and safety of laboratory professionals, ultimately aiming to reduce the incidence of WRMSDs in this occupational group.

## Data Availability

The original contributions presented in the study are included in the article/supplementary material, further inquiries can be directed to the corresponding authors.
